# Survey of Anatomy and Root Canal Morphology of Maxillary First Molars Regarding Age and Gender in an Iranian Population Using Cone-Beam Computed Tomography

**DOI:** 10.22037/iej.2016.8

**Published:** 2016

**Authors:** Mandana Naseri, Yaser Safi, Alireza Akbarzadeh Baghban, Akbar Khayat, Leila Eftekhar

**Affiliations:** a * Department of Endodontics, Dental School, Shahid Beheshti University of Medical Sciences, Tehran, Iran; *; b * Oral and Maxillofacial Radiology Department, Dental School, Shahid Beheshti University of Medical Sciences, Tehran, Iran *; c * Proteomics Research Center, Department of Basic Sciences, Rehabilitation School, , Shahid Beheshti University of Medical Sciences, Tehran, Iran;*; d * Division of Endodontics, Department of Oral Biological and Medical Sciences, Dental School, University of British Columbia, Canada; *; e * Students Research Office, Dental School, Shahid Beheshti University of Medical Sciences, Tehran, Iran *

**Keywords:** Age, Cone-Beam Computed Tomography, Gender, Maxillary Molar, Root Canal Morphology, Tooth Anatomy

## Abstract

**Introduction:**

The purpose of this study was to investigate the root and canal morphology of maxillary first molars with regards to patients’ age and gender with cone-beam computed tomography (CBCT).

**Methods and Materials:**

A total of 149 CBCT scans from 92 (67.1%) female and 57 (31.3%) male patients with mean age of 40.5 years were evaluated. Tooth length, presence of root fusion, number of the roots and canals, canal types based on Vertucci’s classification, deviation of root and apical foramen in coronal and sagittal planes and the correlation of all items with gender and age were recorded. The Mann Whitney U, Kruskal Wallis and Fisher’s exact tests were used to analyze these items.

**Results:**

The rate of root fusion was 1.3%. Multiple canals were present in the following frequencies: four canals 78.5%, five canals 11.4% and three canals 10.1%. Additional canal was detected in 86.6% of mesiobuccal roots in which Vertucci’s type *VI* configuration was the most prevalent followed by type *II* and *I*. Type *I* was the most common one in distobuccal and palatal roots. There was no statistically significant difference in the canal configurations in relation to gender and age as well as the incidence root or canal numbers (*P*>0.05). The mean tooth length was 19.3 and 20.3 mm in female and male patients, respectively which was statistically significant (*P*<0.05). Evaluation of root deviation showed that most commonly, a general pattern of straight-distal in the mesiobuccal and straight-straight for distobuccal and palatal roots occurred. In mesiobuccal roots, straight and distal deviations were more dominant in male and female, respectively (*P*<0.05). The prevalence of apical foramen deviation in mesiobuccal and palatal roots statistically differed with gender.

**Conclusion:**

The root and canal configuration of Iranian population showed different features from those of other populations.

## Introduction

For proper diagnosis and endodontic treatment, knowledge of root and canal morphology is required [1]. One of the reasons of endodontic treatment failure is the inability to detect, debridement and obturate all canals [2]. So, it is a necessity for clinicians to be aware of root canal configurations and anatomic variations [3].

The pulp canal system is complex and canals may branch, divide and rejoin. The clinician must be familiar with the various anatomies of the root canal. Vertucci *et al.* [4, 5], identified eight canal configurations. The most complex root and canal morphology of the maxillary dentition belongs to first molars. Maxillary first molars have been investigated in many studies and most of them have reported 3 roots and 4 canals [3]. The incidences of second mesiobuccal canal (MB2) is estimated in more than 50% of the samples and other variations include distobuccal and palatal roots with more than 1 canal [3].

Morphological variations in root canal anatomy due to ethnicity and genetic differences have been reported in many studies [6-9]; therefore, it is required to identify root canal anatomy of different populations for successful endodontic treatment [10].

Cone-beam computed tomography (CBCT) scanning was introduced to endodontic field in 1990 [11]. This three-dimensional (3D) imaging technique has different applications in endodontics such as diagnosis of periapical lesions due to pulpal inflammation, assessment of root canal morphology [12], localizing a broken endodontic instrument, removal of root fillings, detection of root fractures and analysis of internal and external root resorption [10, 13, 14]. In addition to diagnostic accuracy, CBCT does not damage the tooth structure as most other *in vitro* techniques such as clearing and sectioning do. Also, it saves time during laboratory assessments compared to staining and clearing techniques [10]. CBCT can also be complemented with personal data such as gender, age and tooth position, which may be important in preoperative assessment of root canal morphology for RCT [2].

The aim of this study is to evaluate root and canal morphology of permanent maxillary first molars regarding age and gender in a selected Iranian population using CBCT.

## Materials and Methods

A total of 250 CBCT images of the maxillary first molars were attained from archives of dental imaging center of Shahid Beheshti Dental School in 2014. Images were selected according to the following criteria: molars with fully erupted and matured apices, absence of apical periodontitis, internal/external root resorption, root canal fillings, posts or crown restorations and root fracture or cracks. 

**Figure 1:**
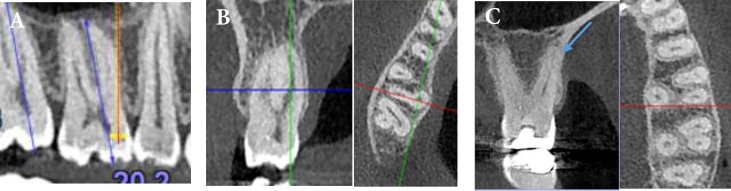
*A)* Calculating the tooth length in panoramic view; *B)* Fused distobuccal and palatal roots; yellow arrow denotes fusion in axial plane, and green one denotes fusion in coronal plane; *C)* A maxillary first molar with 3 separate roots; red arrow denotes second mesiobuccal canal in axial plane; Blue arrow denotes additional canal in palatal root, type *V* of Vertucci’s classification

The CBCT images were taken using NewTom VGi (QR SRL Company, Verona, Italy) with the following parameters: 8×12 cm field of view (FOV), 200 µm voxel size, and 110 kVp and exposure time of 3.6 s. The CBCT images were analyzed with NewTom NNT software version 5.3 (Quantitative Radiology, Verona, Italy). Serial axial, coronal, and sagittal views of CBCT images were examined by carefully rolling the toolbar from the pulp chamber to the apex by a senior dental student and an endodontist independently until an agreed diagnosis was reached for each case. The following anatomic features were recorded: tooth length from the mesiobuccal cusp tip to the apex of the mesiobuccal root on the long axis (Figure 1), number of roots, presence of root fusion, root deviation in coronal and axial plane, number of canals in each root, canal configurations according to Vertucci’s classification, and apical foramen deviation from anatomical apex in coronal and axial plane (Figure 1). Descriptive statistics (age and gender) were also recorded. 

The prevalence of 3-rooted maxillary first molars was calculated. The sample size was determined to be at least 144 teeth, with 95% confidence interval. Independent and paired-t tests were used to analyze the differences of tooth length by gender and age. The Mann Whitney and Kruskal Wallis tests were used for assessing the number of canals and the relation to gender and age. The Fisher’s exact test was used for analyzing the root and apical foramen deviation, canal configuration and root fusion and their correlation with age and gender. Statistical analysis was performed using the SPSS software (SPSS version 21.0, SPSS, Chicago, IL, USA). The level of significance was set at 0.05.

## Results

Two hundred and fifty CBCT scans were examined for inclusion criteria. One hundred and forty nine images of maxillary permanent first molars met all the criteria, including 80 right and 69 left teeth, from 92 (67.1%) female and 57 (31.3%) male patients with a mean age of 40.5 years (Table 1)

***Tooth length***

The mean tooth length was 19.3 and 20.3 mm for female and male patients, respectively. There was a statistically significant difference (*P*=0.001), whereas the difference was not significant with age (*P*=0.296).

***Prevalence of root numbers and fusion***

The prevalence of three roots was 100% in female and male patients. In most cases (98.7%) three roots were separate, but a few (1.3%) had fusion of the roots which was not related to gender and age (*P*=0.620 and *P*=0.289, respectively).

***Prevalence of roots deviation***

In coronal view, almost all three roots were straight, followed by buccal and palatal deviations. But in sagittal plane, the most prevalent deviations were distal, straight and mesial for mesiobuccal root and straight, distal and mesial ones for distobuccal and palatal roots. The deviations of roots did not statistically differ with gender and age except for mesiobuccal root in sagittal plane in which the prevalence of straight root and distal deviations were higher in men and women, respectively (*P*=0.003). Table 2 shows the distribution of root deviation in both coronal and sagittal plane.

***Number of canals***

Tables 3 and 4 show the frequency and percentage of the root canal numbers according to gender and age. The most frequent pattern was four canals (78.5%) followed by five (11.4%) and three canals (10.1%). In 79.9% of teeth four orifices and in 20.1% of teeth three orifices were present at floor of the pulp chamber. Although the number of canals decreases with age, but were not significantly correlated with gender or age of the patients (*P*=0.659 and *P*=0.186, respectively).

***Vertucci classification of canal patterns***

Table 5 shows the frequency of different canal types in the maxillary first molars. Although type *V* canal configuration was seen in the distobuccal and palatal roots, type *I* was the most prevalent in 89.9% of distobuccal and 96.6% of the palatal roots. In the mesiobuccal root, type *VI* canal configuration was the most common in both female (34.8%) and male (36.8%) patients, followed by type *II*, *I*, *IV* and *V*. Type *VII* and *VIII* were not observed. The canal configuration of maxillary first molars did not statistically differ with gender and age (*P*=0.679) and 0.446 for mesiobuccal, (*P*=0.883) and 0.378 for distobuccal, (*P*=0.371) and 0.743 for palatal roots.

***Apical foramen deviation from anatomical apex***

Tables 6 and 7 show the deviation of apical foramen from anatomical apex in coronal and sagittal plans. Mesiobuccal apical foramen location in coronal plane was central (40.3%), buccal deviation (13.4%) and central-central (12.8%) (Two apical foramen) and in sagittal plane was distal (62.4%), central (30.2%) and mesial deviation (6.7%). Distobuccal and palatal apical foramina location in coronal plane were central (50.3-50.3%), buccal (26.2-27.5%), palatal (13.4-18.8%) and in sagittal plane were central (36.2-49%), distal (32.2-34.2%) and mesial deviation (31.5-16.8%), respectively. 

The location of mesiobuccal apical foramen in coronal plane was correlated with patient gender (*P*=0.041), in which prevalence of buccal and central-buccal apical foramen was higher in female patients whereas central-central one was more common in men. Also prevalence of central and mesial apical foramen of palatal canal were higher in female and male patients, respectively (*P*=0.034). There was no significant relation between the location of mesiobuccal apical foramen in sagittal plane, distobuccal one in sagittal and coronal plane and palatal one in coronal plane with gender or age.

## Discussion

This study was the first study in an Iranian population which assessed root and canal morphology of maxillary first molars in relation to age and gender. Knowledge of root canal anatomy and morphology facilitates detection of all canals during root canal treatment. Information such as age and gender of the patients which may be important for pretreatment assessment of the patient have been analyzed in few studies [2]. 

Many studies have examined root and canal morphologies using various methods such as sectioning [15], canal staining and tooth clearing [16-18], modified canal staining and clearing [8], *in vitro* endodontic access cavity with radiography and instruments [19], *in vitro* macroscopic examination[20], *in vivo* root canal therapy with magnification [21], conventional radiography techniques [22], contrast medium-enhanced radiography [23] and cone-beam computed tomography (CBCT) scanning [1].

In comparison to modified canal staining and clearing technique, CBCT is accurate in identifying root canal systems [24, 25]. For detection of the second mesiobuccal canal, the results showed that CBCT scanning is a reliable method compared to the gold standard (sectioning) [26]. Another study stated that micro-computed tomography (µCT) of the canal counts were not different from CBCT results but significantly different from digital periapical radiographies in detection of the extra canals in the mesiobuccal roots of maxillary molars [27]. The advantages of CBCT in comparison with CT are lower radiation dose, reduced exposure time, lower costs and higher accuracy. Furthermore, CBCT measurements are accurate, according to isotropic voxels [28]. In comparison to modified canal staining and clearing technique, CBCT is accurate in identifying root canal systems [24, 25].

**Table 1:** Distribution of maxillary first molars according to patient age

**Age (year)**	**10-20**	**20-30**	**30-40**	**40-50**	**50-60**	**60-70**	**Total**
**Number (%)**	9 (6.0)	42 (28.2)	36 (24.2)	24 (16.1)	21 (14.1)	17 (11.4)	149 (100)

**Table 2:** Roots deviation of maxillary first molars in coronal and sagittal views

**Root**	**Straight-Straight**	**Straight-Distal**	**Straight-Mesial**	**Buccal-Straight**	**Buccal-Distal**	**Buccal-Mesial**	**Palatal-Straight**	**Palatal-Distal**	**Palatal-Mesial**	**Total**
**Mesiobuccal**	18.8	63.1	0.7	3.4	10.1	0	0.7	0	3.4	100
**Distobuccal**	43.0	20.1	17.4	7.4	4.0	5.4	1.3	1.3	0	100
**Palatal**	58.4	8.1	0.7	20.1	4.0	1.3	7.4	0	0	100

**Table 3:** Distribution of number of canals in maxillary first molars according to gender

**Gender **	**Number (%)**			**Total**
	**3**	**4**	**5**	
**Female**	92 (100)	11 (12)	70 (76.0)	11 (12)
**Male**	57 (100)	6 (10.5)	47 (82.5)	4 (7.0)
**Total**	149 (100)	17 (11.4)	11 (78.5)	15 (10.1)

For detection of the second mesiobuccal canal, the results showed that CBCT scanning is a reliable method compared to the gold standard (sectioning) [26]. Another study stated that micro-computed tomography (µCT) of the canal counts were not different from CBCT results but significantly different from digital periapical radiographies in detection of the extra canals in the mesiobuccal roots of maxillary molars [27]. The advantages of CBCT in comparison with CT are lower radiation dose, reduced exposure time, lower costs and higher accuracy. Furthermore, CBCT measurements are accurate, according to isotropic voxels [28]. 

Results of tooth length assessment showed the mean of 19.3 and 20.3 mm for female and male respectively, which is correlated with gender. In anatomical literature, the mean of total tooth length is stated a 20.1 mm [29], which is somehow similar to our findings.

This study found that 1.3% of maxillary first molars among Iranian patients had fused roots. This result was consistent with findings in North American (%0.9) [30] and to a lesser extent, to two other studies on Iranian (2.4%) [10] and Chinese (2.71%) [2] populations. However, previous studies on Burmese [31] and Thai [32] populations, found three separate roots in all maxillary first molars. These differences highlight the influence of ethnic background on root morphology.

All of maxillary first molars had three roots, which was consistent with a previous Korean study [33] and a survey in Hamadan on Iranian population [34]. Incidence of root numbers did not differ with gender and age which is consistent with the results shown in southeastern Turkish population [1].

Root deviation of all three roots in coronal plane was straight (the most frequent), buccal and palatal (the least frequent). In sagittal plane the most common deviations of mesiobuccal root were distal, straight and mesial, and in distobuccal and palatal roots were straight, distal and mesial ones.

The prevalence of additional canals in maxillary first molars have been reported by many studies. In this study the most frequent pattern was four canals, followed by five and three canals, but there were four and three orifices at pulp chamber floor in 79.9% and 20.1% of teeth, respectively. The high frequency (86.6%) of additional mesiobuccal canal is largely consistent with findings of a Brazilian study [35] (86.1% in right molar and 91% in left molar), but higher than Korean [33] (70.5%) and North American (68.2%) population [30]. Although in older patients, frequency of additional canal in mesiobuccal roots was lower, it was not significantly correlated with age or gender in this study. In the Brazilian population older age (*i.e.*, 51-70 years) was associated with fewer additional canals [35] and in Chinese population patients aged 20 to 30 years showed a higher prevalence of additional mesiobuccal root canals which did not differ with gender [2]. In Korean population the frequency of MB2 canals decreased with age but there was not a significant relationship between its incidence and gender [36].

**Table 4:** Distribution of number of canals in maxillary first molar according to age

**Number of canals **	**Age (year)** ** (%)**						**Total**
	**10-20**	**20-30**	**30-40**	**40-50**	**50-60**	**60-70**	
**Three **	2 (22.2)	15 (10.1)	1 (2.8)	1 (4.2)	5 (23.5)	4 (23.5)	15 (10.1)
**Four **	7 (77.8)	117 (78.5)	33 (91.7)	20 (83.3)	13 (61.9)	11 (64.7)	117 (78.5)
**Five **	0 (0)	17 (11.4)	2 (5.6)	1 (12.5)	3 (14.30	2 (11.8)	17 (11.4)
**Total**	9 (100)	149 (100)	36 (100)	24 (100)	21 (100)	17 (100)	149 (100)

**Table 5:** Frequency distribution and percentage of Vertucci classification of canals according to gender in maxillary first molar

**Canal type **	**Mesiobuccal root N (%)**			**Distobuccal root N (%)**			**Palatal root N (%)**		
	**Female**	**Male**	**Total**	**Female**	**Male**	**Total**	**Female**	**Male**	**Total**
***I*** ** (1) **	12 (13)	8 (14)	20 (13.4)	83 (90.2)	51 (89.5)	134 (89.9)	90 (97.8)	54 (94.7)	144 (96.6)
***II*** ** (2-1) **	29 (31.5)	20 (35.1)	49 (32.9)						
***III*** ** (1-2-1) **	2 (2.2)	0 (0)	2 (1.3)						
***IV*** ** (2)**	10 (10.9)	7 (12.3)	17 (11.4)						
***V *** **(1-2) **	7 (7.6)	1 (1.8)	8 (5.4)	9 (9.8)	6 (10.5)	5 (10.1)	2 (2.2)	3 (5.3)	5 (3.4)
***VI*** ** (2-1-2) **	32 (34.8)	21 (36.8)	53 (35.6)						

**Table 6:** Frequency distribution of apical foramen deviation from anatomical apex in coronal plan

	**Central**	**Buccal**	**Palatal**	**Central-Central**	**Central-Buccal**	**Central-Palatal**	**Buccal-Palatal**	**Palatal-Palatal**	**Buccal-Buccal**	**Total**
**Mesiobuccal N (%)**	60 (40.3)	20 (13.4)	15 (10.1)	19 (12.8)	6 (4.0)	18 (12.1)	9 (6.0)	2 (1.3)	0 (0)	149 (100)
**Distobuccal N (%)**	75 (50.3)	39 (26.2)	20 (13.4)	1 (0.7)	2 (1.3)	7 (4.7)	5 (3.4)	0 (0)	0 (0)	149 (100)
**Palatal N (%)**	75 (50.3)	41 (27.5)	28 (18.8)	0 (0)	0 (0)	1 (0.7)	3 (2.0)	0 (0)	1 (0.7)	149 (100)
**Mesiobuccal N (%)**	60 (40.3)	20 (13.4)	15 (10.1)	19 (12.8)	6 (4.0)	18 (12.1)	9 (6.0)	2 (1.3)	0 (0)	149 (100)

**Table 7:** Frequency distribution of apical foramen deviation from anatomical apex in sagittal plan

	**Central**	**Distal**	**Mesial**	**Central-Distal**	**Total**
**Mesiobuccal N (%)**	45 (30.2)	93 (62.4)	10 (6.7)	1 (0.7)	149 (100)
**Distobuccal N (%)**	54 (36.2)	47 (31.5)	48 (32.3)	0 (0)	149 (100)
**Palatal N (%)**	73 (49.0)	51 (34.2)	25 (16.8)	0 (0)	149 (100)

The results of this study indicated that maximum variations in canal anatomy was in the mesiobuccal canal which was consistent with previous studies [10, 32, 37]. In Thai [32], Indian [28], Korean [36] and North American [30] populations the most common canal type for mesiobuccal roots were Vertucci’s type *I* and type *IV*. In the present study, type *VI* (35.6%) and type *II* (32.9%) were the most common types for this root, which was different from another study in Iranian population with dominancy of type *I* and type *VI* (7). The differences among present study with others may be explained by differences in ethnicity, method of evaluation and sample size. Variations in additional canals in distobuccal and palatal roots has been less frequently observed, so that type *I* was the most common one, but type *V* was also seen. It means that although there is one orifice in distobuccal or palatal roots, they may have two canals. This is consistent with some previous case reports in Iranian populations [38, 39]. No significant correlation was observed between root canal system anatomy and gender or age.

The most common location of mesiobuccal root apical foramen in coronal and sagittal plane was central (40.3%) and distal (62.4%), respectively. In most cases apical foramen of distobuccal and palatal roots were central in coronal and sagittal plane. Martos *et al.* [40] reported that in the maxillary molars the most frequent position of apical foramen was central (38%) and the most frequent deviations were to the lingual and buccal positions (21 and 19.2%) followed by mesial and distal (11.1 and 10.7%). Green [41], also reported that in mesiobuccal roots of maxillary molars in 40% of teeth the major apical foramen was directly on the apex and also it was 27% and 31% for distobuccal and palatal roots.

## Conclusion

In the present study the mean tooth length was higher in men and a greater/lesser deviation of the apical foramen from anatomical apex in mesiobuccal and palatal roots was also seen in males. Although in older patients, frequency of additional canal in mesiobuccal roots was lower, it was not significantly correlated with age in this study. Of the limitations of this study was uneven ethnic/racial distribution of the subjects.
